# Hospital and Institutional News

**Published:** 1915-05-22

**Authors:** 


### HOSPITAL AND INSTITUTIONAL NEWS.

### MEDICAL PRISONERS OF WAR.

ON more than one occasion THE HOSPITAL has advocated the view that medical men who happen to be taken prisoners of war should be released at the earliest possible moment. From an answer recently given by Sir Edward Grey in the House of Commons it appears that some negotiations on this point, through the medium of the American Ambassador, have been in progress between our own Government and that of Germany. Both desire, it would seem, to accommodate their practice to the humane regulations of the Geneva Convention, but in certain conditions it would seem that detention may be justified for quite sufficient reasons. For example, in the case of extensive illness among prisoners of their own nationality it would not be unreasonable that medical officers should be retained. This state of matters has actually arrived, for the German Government points out that typhus has been prevalent among the Russian prisoners and that the Russian doctors are more accustomed to deal with the disease than are the Germans; hence, not unnaturally, these doctors are detained. To this principle Sir Edward Grey gave his assent, adding the qualification that while such detention for work actually in hand must be allowed, it must be understood that the permission did not extend to the use of doctors in new and different circumstances.

### VISITS TO THE WOUNDED.

ONE of the most practical ways of adding to the comfort of the wounded in hospital was the establishment of a fund out of which travelling expenses could be provided for the relatives of the wounded men who lived at a distance. It is obvious how much prized is the privilege of seeing sons, brothers, perhaps even sweethearts, when they return wounded from the Front. It has now been suggested, however, that large numbers of the men cannot receive these visits because their friends are so far away. Distance, no doubt, may make the visits few in number and brief, notwithstanding the accommodation that has often been arranged for the friends, by which they can stay a night or two. But is the further statement altogether a happy one, that London residents should organise a system of callers? The matter has two sides. The military regulations are understood to allow visiting only to relations or personal friends. From the point of view of the wounded, visits from strangers will be acceptable or the reverse according to the individual character of the patient. But there is also the hospital side of the question to be considered. The most sympathetic committee realises that visitors are somewhat of a problem, and that if the necessary work of a hospital is not to suffer it is necessary to limit them to certain times and to prescribe regulations for their admission. An army of organised callers, therefore, particularly when they do not know any of the patients personally, presents very obvious difficulties, and though a place may be found for them, it will have to be within strict limits, and only after every effort has been made to prevent distance and long journeys from keeping the wounded and their own personal friends apart. Until it has been shown that the facilities offered to relatives are incapable of useful extension, the visits of strangers must not be rashly encouraged.

### HOSPITALS AND THE SPIRIT DUTY.

AT the time of writing it seems probable that there will be no increased duty on spirit used in hospitals and in the manufacture of spirituous medicinal compounds generally. But since the decision of the Chancellor of the Exchequer to drop his original proposal to double the tax on spirit and to substitute a measure providing for the compulsory bonding of spirits for a period of three years, the position of medicinal spirits has been very uncertain. The Immature Spirits (Restriction) Bill in the form in which it was introduced in the House of Commons contained a provision which would have enabled manufacturing chemists to obtain new spirit, and to utilise it in the preparation of tinctures, etc., but such spirit would have been subject to a surtax which would have been equivalent to about three shillings a gallon on rectified spirit, and would have increased the cost of rectified spirit tinctures by about fivepence per pound. Compared with the effect of doubling the spirit duties the alternative impost would have been small, but since it cannot possibly help the defence of the realm to put an extra tax on hospitals, there ought not to be the slightest surtax on medicinal spirit. It is satisfactory to know that the Chancellor of the Exchequer and the Attorney-General have both assured the House of Commons that there will be no increased tax on spirituous medicines, and it may, therefore, be taken for granted that some way out of the difficulty will be found.

### SIR EDGAR SPEYER AND THE KING'S FUND.

THE anti-German feeling aroused by the publication of the report of Lord Bryce's Committee on German atrocities, and by the sinking of the *Lusitania*, has placed naturalised British subjects of German origin in a very unpleasant situation. Most of those in prominent positions have publicly renewed their expressions of loyalty, but hospital workers will have noted with interest the different action of Sir Edgar Speyer, who has retired from all his public posts. Not only has he asked Mr. Asquith to accept his resignation as a Privy Councillor and to revoke his baronetcy, but he has resigned from the Council of King Edward's Hospital Fund, and from the presidency of the Poplar Hospital.

### THE VALUE OF THE FELLOWSHIP.

MOST hospitals insist upon certain special technical qualifications as necessary for anyone seeking a staff appointment, and on the surgical side the demand is usually for a Fellowship of one or other of the Colleges of Surgeons. In London the English College is generally essential, and every now and again a protest against this exhibition of Metropolitan narrowness, as it is described, is formulated by the Irish Fellows. Quite recently a vacancy was declared at one of the Dublin Hospitals, and candidates were informed that they must possess either the Irish or the English Fellowship, whereupon, not unnaturally, a voice from Scotland exclaims against exclusion by those who preach liberality to the London hospitals. The real position is that the value of these Fellowships is not surgical and technical but disciplinary. The holders are under a certain degree of collegiate government, and it is not unreasonable that colleagues on one and the same staff should in this respect be in an identical position. Hence in London the demand is for the English College, in Dublin for the Irish one, and similarly in Edinburgh and Glasgow. The notion that a man is a great or even a competent surgeon because in his youthful days he managed to pass an examination, possibly by the skin of his teeth, and after much coaching and forced feeding, dwells only in simple minds.

### IS A "WINKLE" A FISH?

This is a question which has recently been propounded to a Divisional Court, and it seems to carry as much legal difficulty as did the much-debated problem, What is a sardine? The discussion was originated in connection with a charge under the Larceny Act, which provides penalties for any person taking "fish" in private waters. The defendant had collected winkles from certain mud flats, and at least some of his trophies were snatched from the water. When the charge was heard the magistrate convicted the accused on the ground that in the eye of the law the winkle is undoubtedly a "fish." In due course an appeal was carried to the Divisional Court, and the judges upheld the decision. The winkle has, therefore, been placed in a proud eminence, and may disdain the efforts of the zoologist to condemn him to a much more lowly rank. What, it may be wondered, would the Divisional Court say to the whale? One of the counsel argued that a "fish" must be a vertebrate, which, of course, would readily accommodate the whale, but he added that a "fish" must be capable of being taken by "angling," and here certainly the monster would escape. The art of definition is a difficult one to pursue with success, and in the recent decision none of the judges proposed to say what is a "fish," but only that the law did not exclude the winkle from this category.

### SHELLS AS PROPHYLACTICS.

Much earnestness has been put into the claim for more munitions in order that we may destroy our enemies, but a writer in the *United Service Magazine* ingeniously argues that the necessity is equally great as a means of preserving the health of our own men. The argument is that the freedom from extensive epidemic disease which has existed hitherto cannot be expected to continue. The seasonal changes, the dust, the flies, added to the abundant organic matter, buried or not buried, will produce the usual results, and the trenches will become pest-houses. To get out of them is then a necessity. This means an advance of the Allied Armies. But to advance into the German trenches will be no improvement; on the contrary, it is likely to prove the proverbial leap out of the frying-pan into the fire. Therefore it is free and large and commanding movement which is necessary. And to attain this munitions and more munitions are required. Thus what is declared on authority to be a military necessity is found equally to be demanded by sanitary science.

### WHAT IS A STILL-BORN CHILD?

For many reasons there are often difficulties in determining from the examination of the dead body of an infant whether or not in the legal sense it has been "born alive." The lawyers will not allow such birth unless there is proof that the child had an independent existence after being fully delivered from the body of its mother. Hence, even if the medical witness can say from an examination of the lungs that the child has breathed, he will be unable to say whether the respiration took place before or after complete delivery. "Live-birth," in the legal sense of the term, is therefore hard to establish except by a witness who was present at the birth, and in charges of infanticide there is as a rule no such witness. But whatever uncertainty may exist on this point there ought to be no doubt that a child which, after delivery, shows signs of life is not still-born. The signs may be of the most restricted order—a faint cry, a respiratory effort, a cardiac impulse, a muscular movement—but they are sufficient to establish the legal existence of the individual. In such circumstances the child has come "under the King's peace," and though his life last but a moment or two his birth and death must each be duly registered. The question legally is not, Was the child viable? but, as a matter of fact, Did it show signs of independent life? From a recent inquest it would appear that even yet all practitioners are not alive to this position of affairs. In the case in question the doctor admitted that he had given a certificate of still-birth because from his calculations he concluded that the birth had taken place thirty days short of viability, and, therefore, the child could never have "lived properly." In this instance no unfortunate consequences followed except to the doctor himself, but there have been cases in which the direction of large parcels of property has depended on the issue whether a given infant was or was not still-born. The answer, we repeat, is determined not by any question of viability or non-viability, but merely by the plain matter of fact that the infant did or did not show signs of life after delivery was complete.

### LISTER'S HOUSE IN LONDON.

In one sense Lister, of all men who have served their fellows, is little in need of formal tablets and tributes and memorials. His work has not only an enduring quality, but it is in daily and hourly operation and application. The horrors of the battlefield and the pathos of the ambulance train and the military hospital are in all conscience acute enough, but they would have been infinitely worse but for the sustained and penetrating work of Lister. Curiously enough, in view of the universal fame he gained, his local interests were scattered over many centres. Born and educated in England, it was yet in Glasgow that his famous system was devised and applied; later he was Professor in Edinburgh, and subsequently moved to London, where, such was the prejudice of the hour, he received in certain professional quarters anything but a worthy welcome. In his old age his West-End residence was often pointed out to the visitor to the Metropolis, and it is meet that future generations should be reminded of it. To effect this the London County Council has determined that a simple tablet shall be placed on the house, No. 12 Park Crescent, Portland Place, W., announcing that here for a number of years Lister lived.

### CONFERENCE ON THE FEEBLE-MINDED.

The National Association for the Feeble-Minded will hold its annual conference at the Guildhall Council Chamber, London, on June 25. The subject of the conference concerns the methods of examination best adapted to ascertain the presence, or otherwise, of mental defect. Sir Bryan Donkin, M.D., will preside. At the morning session four papers will be read on various aspects of the detection of mental deficiency in children, including one by Dr. W. B. Drummond, medical officer Children's Hospital, Edinburgh, on "Binet Simon Tests as a Means of Grading Mental Defectives under the Mental Deficiency Act." At the afternoon session six papers will be read on such subjects as the classification of the mentally defective from administrative and legal standpoints, the value of a uniform examination, practical difficulties in regard to medical examination under the Mental Deficiency Act, and the characteristics and identification of the feeble-minded criminal. An exhibition of the work done by the inmates of institutions for the mentally defective will also be on view. Admission is by ticket, which can be obtained from the secretary at the Association's offices, Denison House, 296 Vauxhall Bridge Road.

### THE BELVIDERE HOSPITAL INQUIRY.

The Commissioners instructed by the Local Government Board to hold an inquiry into the death of a boy in Belvidere Hospital, Glasgow, have issued their report. To put it briefly, they state that the evidence points to the disease having been diphtheria; and that Dr. Alexander Johnston, the physician-superintendent, was not justified, on the examination he made, in discarding the previous diagnosis; and that they think he failed in his duty by allowing, without remark, a young assistant to treat for diphtheria a case which he had determined was not diphtheria. Lastly, they found that there was no undue delay in removing the patient to the hospital. Dr. Johnston characterises the four conclusions as "contradictory in terms," "misleading," "inaccurate," and "gratifying." He adds in a letter to the Town Clerk that the responsibility of his post lends weight to the decisions he arrives at, which should be considered final judgments relative to classification and diagnosis of cases, but he fully admits the fallibility of human judgment in the diagnosis of disease. "I have been compelled," he concludes, "in view of the partial report of the Commissioners, against which I have no appeal, to consider my position." He, therefore, has tendered his resignation to the Corporation, under whom he has held the above post for twenty-two years. The Health Committee have recommended its acceptance, and that he be granted an allowance of one year's salary, amounting, with emoluments, to £700.

### STRIKERS AND THE WOUNDED.

A curious situation has arisen in connection with the building of a temporary ward for the wounded at Northampton Hospital, over which delay has occurred through a strike of 1,000 building-trade workers for 1d. an hour increase. After matters had gone so far that 100 wounded soldiers who had been sent to the hospital had to be refused because the new ward was unfurnished, a conference was held, at which the men offered to return to work at the rate of pay existing before the strike, provided that any difference between this rate and that fixed after a settlement were made retrospective. As the employers' representatives were not authorised to commit themselves, nothing came of it. But the carpenters and joiners, who are naturally those most involved in the work, stated that if the above terms were not accepted by the employers they would do the work for nothing if the contractors would undertake to hand over their wages to the Red Cross Fund.

### A MEDICO-LEGAL POINT.

It is curious that, in spite of history and experience, every now and again a situation arises in which a new problem is presented for solution. Here is one which was recently argued at a meeting of one of the divisions of the Medico-Psychological Association. A patient is certified and is under formal care and control. For reasons which do not touch the issue, the police wish to arrest him. In answer to the protest that the man is insane the police reply is that how far this is true must be determined by the prison medical officer, with whom rests the duty of advising the Court whether the prisoner is or is not fit to plead in answer to the charges made against him. The asylum authorities hold the view that the man's chance of recovery will be prejudiced if he is made to undergo this discipline. In these circumstances ought they to give him up to the police? The experts differ in their answers, but to the non-expert mind there would seem to be no great difficulty in the situation. At least it may be said there is some guidance from the parallel case where a person who is wanted by the police is the subject of injuries not mental but physical. Say a man commits murder and then cuts his own throat short of a fatal result. He is taken to a civil hospital and is cared for by civil surgeons until he can be certified as fit for removal to prison. Then, and then only, is he handed over to the police, because only in these circumstances can the man be transferred without risk to his physical health or even to his life. It is true that during his residence in a civil hospital he is watched by a police officer in order to prevent any attempt at escape. But why, if the circumstances demand it, may not a similar policy be pursued at an asylum? A citizen is, in the eye of the law, innocent until he is proved guilty. It surely cannot be right that by the action of the State the chance of an innocent man's recovery of his reason should be diminished; nor, indeed, is any such penalty prescribed, even in the case of guilt.

### THE NAVY AND MILITARY DOCTORS.

THERE is a general impression that while the Army doctors are deficient in numbers and many of them are overworked, doctors in the service of the Navy are too numerous and have little to do. The suggestion has therefore been made that the senior Service should transfer some of its medical officers to the Army. Some comment of the same order has been made in reference to the consulting physicians and surgeons retained by the Admiralty, and these gentlemen have been pictured as having nothing to do but to pocket their emoluments and at the same time to receive large fees for private practice. The actual state of matters is very different from this. But, leaving this position aside, it has to be remembered that in both Services preparations for possible emergencies have to be made. The great naval fight long expected has not yet taken place, but who shall say that it may not arrive at any moment? And what would kindly critics say of the First Lord if he explained that he had not sufficient doctors to attend to wounded sailors because he had given his medical officers to the Army in Flanders? It is true, of course, that the proportion of killed to wounded has been much higher in the Navy, since a naval battle means sink or be sunk.

### THE LISTER INSTITUTE.

IT will be remembered that the governors of the Lister Institute proposed last year to unite their organisation with that of the Medical Research Committee established under the National Insurance Act. The proposal excited much comment and led to a rather acute controversy. In the end, in November last, it was considered at a meeting of the members of the Institute and was rejected. There was, however, a general feeling that this vote was not to be accepted as the final word and that the advocates of amalgamation had not exhausted their resources. This view receives some confirmation from an intimation in the recently issued official report in which the statement is made that in existing circumstances there is no intention to ask for a reconsideration of the adverse vote, though this decision is not to be taken as implying its acceptance. When conditions improve it is hoped that further friendly discussion of the position may be possible. Besides other preparations, the Institute during the first eight months of the war delivered to the Army 102,340 doses of tetanus antitoxin, 18,500 doses of diphtheria antitoxin, and 770,000 doses of antityphoid vaccine; also to the Navy 18,300 doses of tetanus antitoxin.

### MEDICAL RECRUITS.

IN these days the announcement of those who have been admitted licentiates or members of the Royal Colleges of Physicians and Surgeons is a matter of public interest in a new and important sense. We are so much becoming habituated to looking at casualty lists that there is a real public satisfaction in learning of the hundred candidates who were admitted last week as members of the Royal College of Surgeons. While the demand continues to outrun the supply, each new batch of qualified medical men is of importance, and the sense that this is so is so general that we are apt to forget the recent cry that the profession was overstocked, notwithstanding the new branches of public medicine which have expanded so much of recent years. That cry, probably never true beyond the sphere of private practice, for the time being has received its death-blow, and each new accession to the ranks is as inspiriting as ordinary recruiting, although the profession of medicine requires as many years of training as the months demanded for the profession of arms.

### MEDICAL OFFICER AND HIS HALF-PAY.

THE Bermondsey Board of Guardians some weeks ago granted leave of absence to Dr. J. T. Macnamara, their medical officer at Ladywell Institution, to accept service in the Royal Army Medical Corps. Whilst deciding to keep open the appointment they qualified their concession by not allowing him "half-pay," in accordance with a resolution adopted at the commencement of the war that all officers joining the Forces should receive "half-pay" during their engagement with the Forces. The reason they decided on this course was that they held that Dr. Macnamara was not entitled to it, as he was only a part-time officer. In a letter to the board, Dr. Macnamara points out that he accepted service on the understanding that the board's resolution applied to him, and that, even under these conditions, he was making a financial sacrifice of close on £100 a year, but by reversing their decision the board were imposing an additional financial sacrifice upon him of £125 a year during the period of the war. He felt confident it could never have been the intention of the board to penalise one of its officers to the extent of over £200 a year for giving his services to the country in the present crisis, and he appealed to the Guardians to review the circumstances. Although the visiting committee decided to take no further action, the board itself would not accept their proposal, but resolved that their resolution granting "half-pay" to those officers who had joined the Forces should apply to Dr. Macnamara. Their decision removes a great injustice.

### A MEDICAL OFFICER'S BRAVERY.

A REMARKABLE instance of bravery on the part of a medical officer at the Front has been announced, the hero of which is Surgeon-Major H. Legh de

Legh, of the 4th Yorkshire Territorials. It is understood that he has been recommended for distinction to the Commander-in-Chief for his courage in rescuing twelve wounded soldiers from a farmhouse used as a dressing-station, which was set on fire by petrol shells. When the shells took effect the soldiers who were severely wounded were in the cellar, when Dr. Legh de Legh, with his stretcher-bearers, at great personal risk went to their aid and succeeded in getting them out just as the house collapsed. They were laid on the ground a short distance away from the blazing house, since there was no other place near by to which they could be taken. It is also gratifying to learn that the doctor, who is a resident at Redcar, has interested himself for over twenty years in the Territorials and Volunteers.

### POOR-LAW MEDICAL OFFICERS' ASSOCIATION AND LADY VISITORS.

At a recent meeting of the Council of this Association a number of questions of policy came up for discussion, and some of them involved features of general interest. Among these questions were several which involved the extent of the obligations incumbent on a medical officer. Thus the query was asked whether a lady visitor had authority to direct an officer to give attendance on some individual or to demand from him a report on a patient under his care. It is hardly surprising to find that in each instance the answer to her ladyship was in the negative. Similarly it was decided that a medical officer cannot be expected to estimate errors of refraction and to prescribe the lenses necessary for their correction. Nor can the clerk of the Guardians demand from him a report on the sanitary state of a house, for plainly this duty falls within the sphere of the medical officer of health. Another discussion of some interest dealt with the relations of Poor-Law medical officers to the Army authorities. In the end it was decided that where such officers accepted commissions in the R.A.M.C. they ought to receive the same salaries as their military *confrères* engaged in similar duties; also that for part-time service there ought to be an addition to the officer's salary in proportion to the labour involved.

### NEW MEDICAL ASSOCIATION IN IRELAND.

The doctors who have been appointed by the Insurance Commissioners to grant certificates for benefits under the Act have determined to form an association for the protection of their personal and professional interests. At the inaugural meeting the following resolution was adopted: "That inasmuch as the additional £40,000 promised by the Chancellor of the Exchequer for medical certification in Ireland has not yet been utilised owing to the failure of the medical profession and the approved societies to agree to a scheme, we, whilst pledging ourselves to work the existing scheme of the Insurance Commission, are anxious to assist in the furtherance of any final settlement of this question in the future, provided always that the present certifiers receive due consideration." The president is Dr. H. F. Powell and the hon. secretary Dr. Philip Purcell.

### DOCTORS AND DENTISTRY.

The Norfolk Insurance Committee has been struggling with a somewhat difficult and delicate issue. The question considered was whether a doctor by the terms of his contract is bound to give an anæsthetic to a patient on his panel list who needs dental treatment. It was admitted that the dental treatment is not part of the doctor's responsibility, but there were not wanting some members who claimed that the anæsthetic is in a very different position. The argument was developed with some ingenuity. It is the patient's ill-health which makes the anæsthetic necessary, and the doctor is bound to apply the needed treatment. To extract teeth is one thing, and to give an anæsthetic is another and entirely different thing. The one event, whichever way the reading be taken, may happen without the other; why, then, should not the responsibility for the two be allotted to a different department? All this ingenuity, however, proved to be in vain, and by a substantial majority the Committee refused to read into the doctors' agreement a bond of which the doctors themselves denied the existence.

### THE WAR AND THE COST OF INSTITUTIONAL TREATMENT.

The Sanatorium Benefit Sub-Committee prepared for submission to the London Insurance Committee at its meeting on May 20 a report relating to the arrangements with the Metropolitan Asylums Board for providing treatment in institutions for insured persons suffering from tuberculosis. Some time ago the Committee decided to enter into arrangements with the Metropolitan Asylums Board with a view to providing treatment at the Downs and Winchmore Hill Sanatoria and other of the Board's institutions at the rate of 29s. 6d. per week per patient. The Board has now drawn attention to the fact that since the outbreak of war serious increases in the prices of food and other supplies, and in wages, have materially affected the cost of maintenance of all inmates in the Board's institutions. The Board states that during the earlier period of the war contracts for supplies were in force; but, following the action taken by the Government and by local authorities throughout the country, the reasonable claims of contractors for extra cost incurred by them in circumstances over which they had no control had been conceded. The Board is therefore asking the authorities concerned to share the extra cost by way of an additional charge of 10 per cent. over a specified period. The Sanatorium Benefit Sub-Committee advises the Insurance Committee to accede to this request, and recommends that a payment to the Board of £2,196 18s. 7d. be authorised in respect of the extra cost of maintenance, owing to the war, for tuberculous insured persons at the institutions named during the period August 5, 1914, to March 31, 1915.

### MENTAL SUPERINTENDENT'S RESIGNATION.

The retirement of Dr. Rowe, medical superintendent of the Borough Mental Hospital, Ipswich, after twenty-six years' service, is to take place in two months' time. Dr. Rowe has resigned as a result of continued ill-health, and a remarkable tribute was paid to him by a member of the visiting committee, who declared that during all these years he did not think there had been a single unfortunate occurrence. Under the provisions of the Asylums Officers' Superannuation Act, 1909, he is entitled to a superannuation allowance.

### MENTAL TREATMENT WITHOUT CERTIFICATION.

In consequence of a Bill recently introduced in the House of Commons some attention is being given to the practice of dealing with borderland and allied cases in Scotland as compared with the rule in operation in England. It seems that in the North cases which are certifiable can be treated under compulsion out of asylums and without certification, so long as the malady from which they suffer is not a confirmed one. Hence cases of temporary mental disturbance may and, as a matter of fact, do escape the brand of insanity, where a patient in an exactly similar position in England would be certified, and thus, though recovering and remaining well, would carry a stigma for the rest of his days. It is sometimes the case that patients whose friends are aware of the conditions ruling north of the Tweed are actually taken to Scotland in order to avoid the penalty of the English procedure.

### FACE-PIECE FOR POISONOUS GASES.

Mr. James Cantlie has devised a simple apparatus having for object both the protection of the eyes and the neutralisation of the inhaled air when the soldier is exposed to such gases as chlorine and bromine. In front of the eyes is uninflammable cellulose material, while a pad covers the mouth and nostrils. Both are parts of a mask which can be secured by tapes, and the pad is filled with sodium hyposulphite and sodium bicarbonate. The moisture of the breath is sufficient to make these salts effective as neutralising agents even if water is not available. The face-piece is made by Messrs. Allen and Hanburys, and the price is 2s. 6d.

### PENSIONERS AND MEDICAL ASSISTANCE.

For some time past the Local Pension Committee of the County of London has been trying to solve a difficult problem in connection with the right of old-age pensioners to medical assistance. At present pensioners frequently refrain from applying to the Poor-Law authorities for medical and surgical assistance because they believe that it will necessarily disqualify them for a pension. As it seemed to the Committee in every way desirable that pensioners should know that such is not the case, they suggested to the Board of Customs and Excise that a short statement of the law should be printed on each pension order book. The Board replied that after careful consideration they regretted that they did not feel able to adopt the suggestion. They gave no reason for their decision, and the Committee accordingly have urged them to reconsider it.

### DRUGS AND OPTIC ATROPHY.

Many practitioners will remember how a few years ago certain organic compounds of arsenic were energetically boomed in the treatment of syphilis and of certain organic nervous diseases. Then the usual preliminary triumphs were followed by warnings of possible disaster, and cases began to be recorded in which optic atrophy had resulted from the treatment. Another "new remedy" seems likely to pass under a similar condemnation. This is optochin, or ethyl-hydro-cuprein. Laboratory experiments showed that this compound has a high bactericidal action upon the pneumococcus, and so it was tried in various pneumococcal infections. Good results were reported from its local use in such infections of the cornea and conjunctiva. Next followed its internal administration in pneumonia, and several authorities spoke favourably of such treatment. Among the reports, however, were instances in which the patients developed amblyopia, though this, it was affirmed, was always a mere temporary experience. A case is now reported in which sudden blindness developed, with optic discs white and atrophic-looking, the whole picture holding out little prospect of much substantial improvement. Such an experience is an alarming one, and seems to justify a verdict once delivered to the effect that the really dangerous practitioner is he who treats old symptoms with new remedies.

### ITALY AND THE DRUG MARKET.

The tone of the drug market has been somewhat quieter, but business is likely to be as active as ever again before long. On the whole prices are well maintained, the advancing tendency continuing in the case of a number of drugs. The probability that Italy will shortly be at war does not appear to have affected materially the prices of drugs obtained from that country. The values of essence of lemon, oil of bergamot, and citric acid are firmly maintained, but there has been no pronounced advance during the past few weeks. Tartaric acid and cream of tartar are in good demand and prices tend higher. Salicylic acid, salicylate of soda, and acetyl-salicylic acid are again dearer, but there is little change in the position of synthetic drugs generally. Potassium permanganate is dearer. Further business has been done in quinine, and the price has an upward tendency. Cod-liver oil is, if anything, a little lower in price, and while it is difficult to foresee the future of this market, it is at least possible that we may see a small decline in prices. The value of ipecacuanha is well maintained. Cardamoms have a slightly lower tendency in value. Oil of cloves is a trifle cheaper. Honey is dearer. The attitude of buyers should be a cautious one, especially in respect of those products the values of which have advanced to such high levels.

